# Simulator-Based Angiography and Endovascular Neurosurgery Curriculum: A Longitudinal Evaluation of Performance Following Simulator-Based Angiography Training

**DOI:** 10.7759/cureus.756

**Published:** 2016-08-29

**Authors:** J. Scott Pannell, David R Santiago-Dieppa, Arvin R Wali, Brian R Hirshman, Jeffrey A Steinberg, Vincent J Cheung, David Oveisi, Jon Hallstrom, Alexander A Khalessi

**Affiliations:** 1 Department of Neurosurgery, University of California, San Diego; 2 Department of Internal Medicine, University of California, Los Angeles; 3 Neuroradiology, University of New Mexico

**Keywords:** endovascular neurosurgery, neurosurgical education, angiography training, neurovascular training, neurosurgical performance evaluation, endovascular coiling, quality improvement, cerebral angiogram, procedural skills, simulator-based angiography

## Abstract

This study establishes performance metrics for angiography and neuroendovascular surgery procedures based on longitudinal improvement in individual trainees with differing levels of training and experience.

Over the course of 30 days, five trainees performed 10 diagnostic angiograms, coiled 10 carotid terminus aneurysms in the setting of subarachnoid hemorrhage, and performed 10 left middle cerebral artery embolectomies on a Simbionix Angio Mentor™ simulator. All procedures were nonconsecutive. Total procedure time, fluoroscopy time, contrast dose, heart rate, blood pressures, medications administered, packing densities, the number of coils used, and the number of stent-retriever passes were recorded. Image quality was rated, and the absolute value of technically unsafe events was recorded. The trainees’ device selection, macrovascular access, microvascular access, clinical management, and the overall performance of the trainee was rated during each procedure based on a traditional Likert scale score of 1=fail, 2=poor, 3=satisfactory, 4=good, and 5=excellent. These ordinal values correspond with published assessment scales on surgical technique.

After performing five diagnostic angiograms and five embolectomies, all participants demonstrated marked decreases in procedure time, fluoroscopy doses, contrast doses, and adverse technical events; marked improvements in image quality, device selection, access scores, and overall technical performance were additionally observed (p < 0.05). Similarly, trainees demonstrated marked improvement in technical performance and clinical management after five coiling procedures (p < 0.05). However, trainees with less prior experience deploying coils continued to experience intra-procedural ruptures up to the eighth embolization procedure; this observation likely corresponded with less tactile procedural experience to an exertion of greater force than appropriate for coil placement.

Trainees across all levels of training and prior experience demonstrated a significant performance improvement after completion of our simulator curriculum consisting of five diagnostic angiograms, five embolectomy cases, and 10 aneurysm coil embolizations.

## Introduction

Simulation has been integrated into medical training for many years, whether in the form of cadaveric dissection, the use of animal models, or synthetic models. Yet, the idea of simulation in modern medicine dramatically evolved with the emergence of the computer during the latter portion of the 20^th^ century. The term "simulation" is now synonymous with computerized virtual reality devices and been effectively introduced in aviation. Though neurosurgery remains a field unforgiving of technical errors, simulation devices remain underutilized due to challenges in three-dimensional (3D) simulation. In contradistinction, endovascular interventions rely on a two-dimensional (2D) angiographic interface and reasonably reproduced haptics of catheter and wire manipulation. Neurovascular surgery, therefore, is a natural target for the development of a robust simulation curriculum.

Simulation in neurosurgery facilitates a zero-risk introduction to skills sets that are often not learned until the senior resident or fellow levels. Simulation is particularly important in the current environment of work hour restrictions and emphasis on patient safety. Given the clear benefits to developing complex motor and planning skills without the potential for adverse patient complications, integration of simulation in neurosurgical education is both rapidly becoming an area of increasing interest for all neurosurgical subspecialties and of critical importance for the future [1-26].

To date, there are only three studies that specifically evaluate the use of virtual simulation in endovascular neurosurgery [1, 23, 25]. The first article to support the role simulation-based training in cerebral angiography was published in 2012 by Fargen, et al. [25]. This study evaluated seven resident-level learners with no prior angiography experience before simulator experience [25]. Fargen, et al. found that in addition to higher post-simulation written and faculty assessments, there was a statistically significant decrease in both procedure and total fluoroscopy time with subsequent trials. In 2013, Spiotta, et al. published a study using a standardized training protocol for a Symbionix angiography simulator [23]. This larger study involved 14 participants over five consecutive trials. This study found that resident-level learners were more likely to make potentially dangerous errors when compared to fellow-level learners. Like the Fargen study [25], Spiotta, et al. also found a statistically significant decrease in procedure and fluoroscopy time with subsequent trials [23]. The most recent study published by Fargen, et al. involved the evaluation of a 120-minute simulator-based training course for 37 participants [1]. As found in the previous two studies, post-test written and practical scores showed a statistically significant increase after simulator training [1, 23].

To date, there are currently no studies evaluating the use of virtual simulation for endovascular neurosurgical interventions, such as aneurysm coilings or mechanical embolectomy. Moreover, the number of simulator sessions needed to establish minimum endovascular competence remains undetermined. The purpose of this study is to establish performance metrics for angiography and neuroendovascular surgery procedures based on longitudinal improvement in individual trainees with differing levels of training and experience.

## Materials and methods

Trainees who agreed to participate were explained the nature and the objectives of the study, and informed consent was formally obtained.

After preliminary didactic instruction, each of the five participants performed 10 nonconsecutive four-vessel diagnostic cerebral angiograms, coiled 10 nonconsecutive carotid terminus aneurysms, and performed 10 nonconsecutive mechanical embolectomies over the course of 30 days on an endovascular simulator. The participants were given clinical scenarios to accompany each procedure and were evaluated based on both periprocedural clinical management and intraprocedural performance.

### Preliminary didactic instruction

All participants underwent 120 minutes of didactic instruction in angiography and endovascular neurosurgery that encompassed macrovascular brachiocephalic anatomy, microvascular cerebral anatomy, macrovascular access, microvascular access, and basic principles of clinical as well as endovascular management of acute ischemia due to large vessel occlusion (LVO) and hemorrhagic stroke due to aneurysm rupture. All participants witnessed one simulated diagnostic angiogram, one simulated carotid terminus aneurysm coiling, and one simulated mechanical embolectomy performed by an experienced operator on the same simulator used for the development of this curriculum prior to independent operation.

### Participants

Five trainees with varying backgrounds with limited endovascular experience were involved in this study. The study included a second-year medical student, one first-year neurosurgery resident, two first-year diagnostic neuroradiology fellows, and one first-year endovascular neurosurgery fellow. The first-year neurosurgery resident and the medical student had never performed a diagnostic cerebral angiogram. Both of the first-year diagnostic neuroradiology fellows had previously participated in less than five total cerebral angiograms. Four of the five participants had never performed any prior coil embolization with the exception of the first-year endovascular neurosurgery fellow.

### Simulator

**Figure 1:**
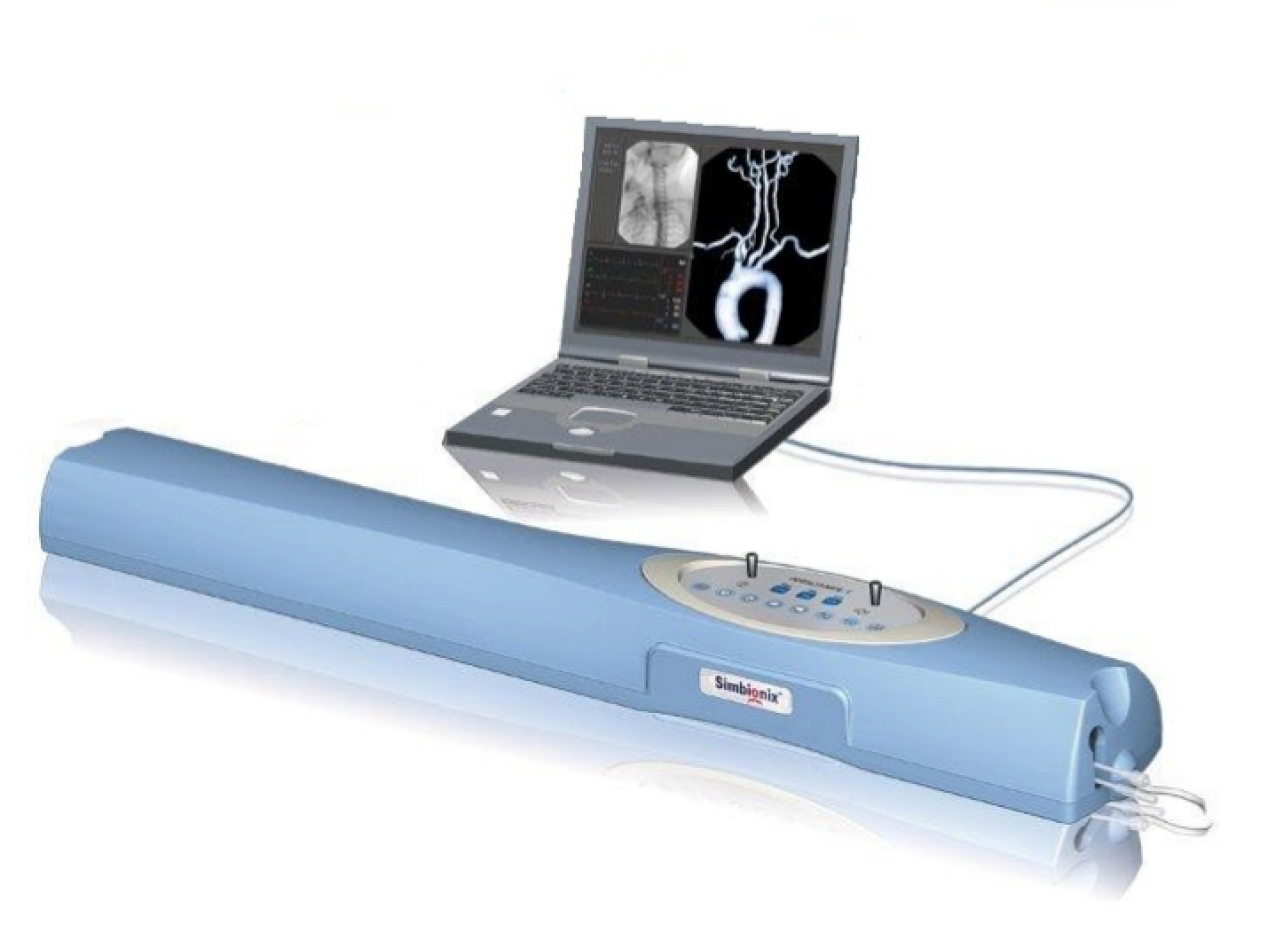
Digital photograph of the Simbionix ANGIO mentor simulator used for this study

One Simbionix ANGIO mentor^TM^ simulator (3D Systems, formerly Simbionix, Cleveland, Ohio, USA) was used for this study (Figure 1). The Simbionix ANGIO mentor^TM^ simulator utilizes actual catheters and wires introduced through a port similar to the diaphragm of an arterial sheath. The wires and catheters engage internal rollers that record both rotational and translational motion, allowing for the simulation of finely controlled application of force vectors required for the performance of angiography and endovascular neurosurgery. A joystick controls the simulated angular positioning of the imaging intensifier relative to the patient, and two separate buttons control the proximity to the patient. A separate joystick controls the bed position. The simulated position of the image intensifier relative to the patient is displayed on a single screen that minimizes the need for use of fluoroscopy. Three separate foot pedals initiate simulated fluoroscopy, roadmap, and digital subtraction angiography. The simulator simulates physiologic responses to aneurysm rupture and ischemic stroke by elevation of blood pressure and heart rate. The simulator allows the operator to administer anticoagulants, antihypertensives, and pressors. The simulator also allows the operator to test activated clotting times.

### Scenarios

For diagnostic angiograms, participants were presented with a scenario of a 52-year-old male with an unruptured left carotid terminus aneurysm found incidentally on a non-contrast computed tomography (NCT) of the head.

For aneurysm coiling, participants were presented with a scenario of a 52-year-old male with a known ruptured left carotid terminus aneurysm, severe headache, non-focal exam, and a Glasgow Coma Scale of 15. The participants were presented with an NCT demonstrating a Fisher Grade II subarachnoid hemorrhage and no hydrocephalus.

For ischemic stroke, participants were presented with a scenario of a 64-year-old female with a National Institutes of Health Stroke Scale (NIHSS) score of 12 for aphasia and right-sided weakness who was last known to be normal four hours prior. The participants were presented with NCT revealing Alberta Stroke Program Early CT score (ASPECTS) score of 10, no hemorrhage, and hyperdense left MCA sign as well as a CT angiogram revealing left M1 segment MCA occlusion.

### Performance evaluation

The evaluation was performed by a neurointerventional attending of greater than two years of experience. Total procedure time, fluoroscopy time, contrast dose, heart rates, blood pressures, medications administered, packing densities, the number of coils used, and the number of stent-retriever passes were recorded for each procedure performed by all participants. Image quality was rated based on alignment, selection digital magnification, the field of view, object to image intensifier distance, and geometric magnification. The absolute value of technically unsafe events, including too little leading wire, rapid forward movements with devices, off screen with devices, uncontrolled loss of access, unobserved movement of devices, unintended catheterization of non-target vessel, forward guide catheter movement without leading wire or catheter, coil deployment in the parent artery, failure to perform angiograms prior to detachment of initial coil(s), failure to perform final angiogram, device outside confines of roadmap, and number of intraprocedural ruptures, were recorded.

**Figure 2:**
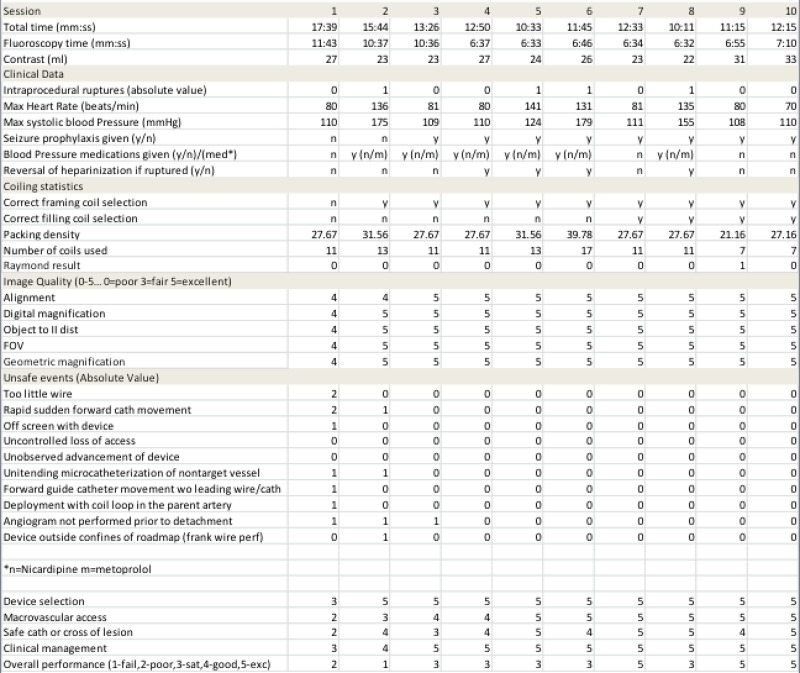
Example scoring sheet Scoring of participants during aneurysm coiling

The trainees’ image quality, device selection, macrovascular access, microvascular access, clinical management, and overall performance of the trainee were rated during each procedure based on a traditional Likert scale score of 1=fail, 2=poor, 3=satisfactory, 4=good, and 5=excellent (Figure 2).

## Results

### Diagnostic cerebral angiography

**Figure 3:**
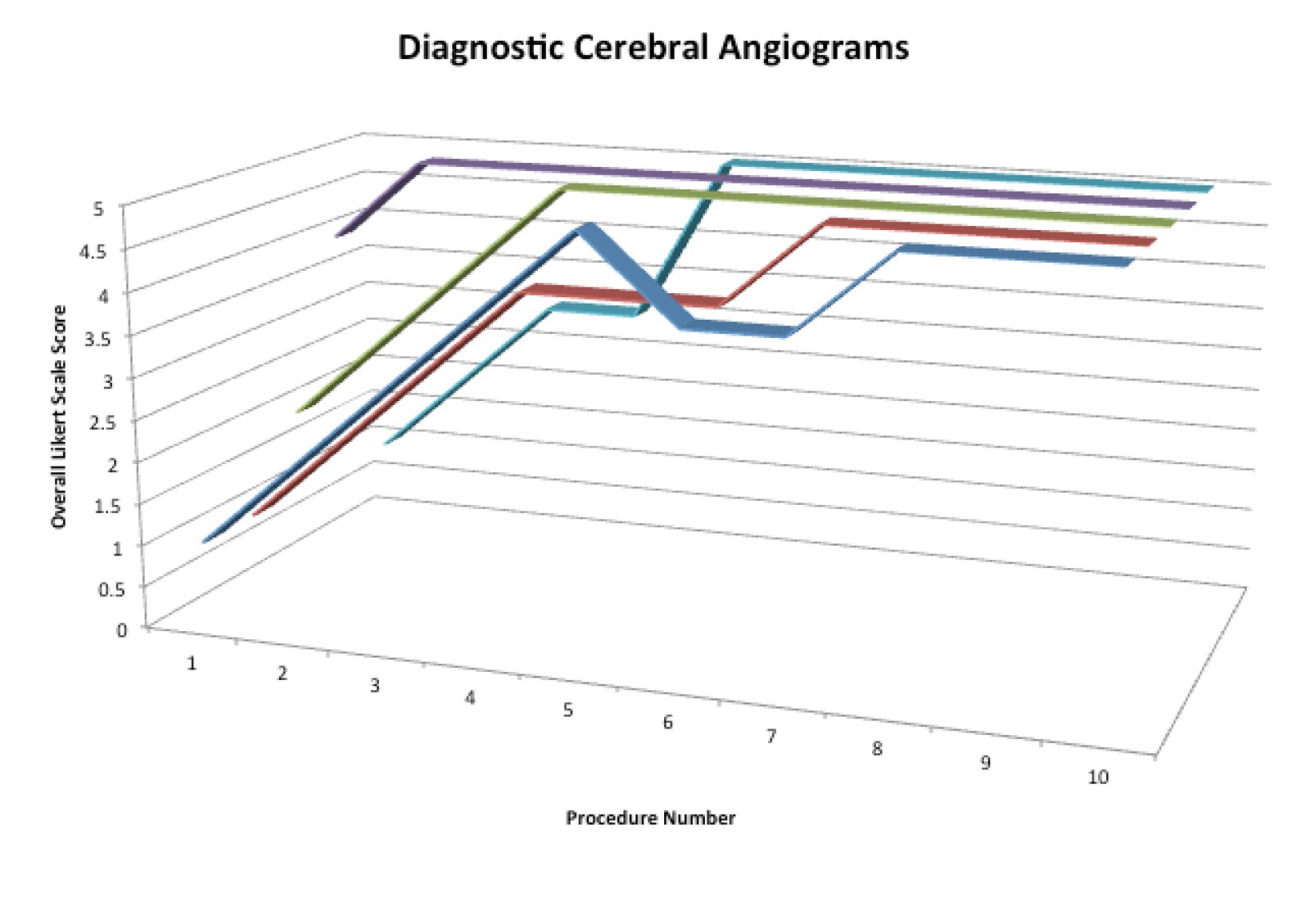
Color-coded 3d line graph of overall Likert Scale performance scores Scores for each participant over the course of performing 10 diagnostic cerebral angiograms. Each participant is displayed in a different color.

After completion of the diagnostic angiography portion of our simulator curriculum, participants demonstrated an 86% reduction in total procedure time, a 75% reduction in fluoroscopy time, and a 68% reduction in contrast utilization. There was a 25% improvement in the image quality. There was a reduction in unsafe techniques from an average absolute value of 4.2 events to zero events. There was a 64% improvement in the overall Likert Scale performance score (Figure 3).

All variables were compared using the analysis of variance (ANOVA) and Tukey’s Honest Significant Difference (HSD) tests to evaluate the significance of each variable between each angiogram. Independent of prior experience, all participants demonstrated statistically significant differences in performance during the first five angiograms (p < 0.05 for all variables). During the last five angiograms, all participants demonstrated improvement, but the differences were not statistically significant on the 95% confidence interval (average p = 0.216).

### Aneurysm coiling

**Figure 4:**
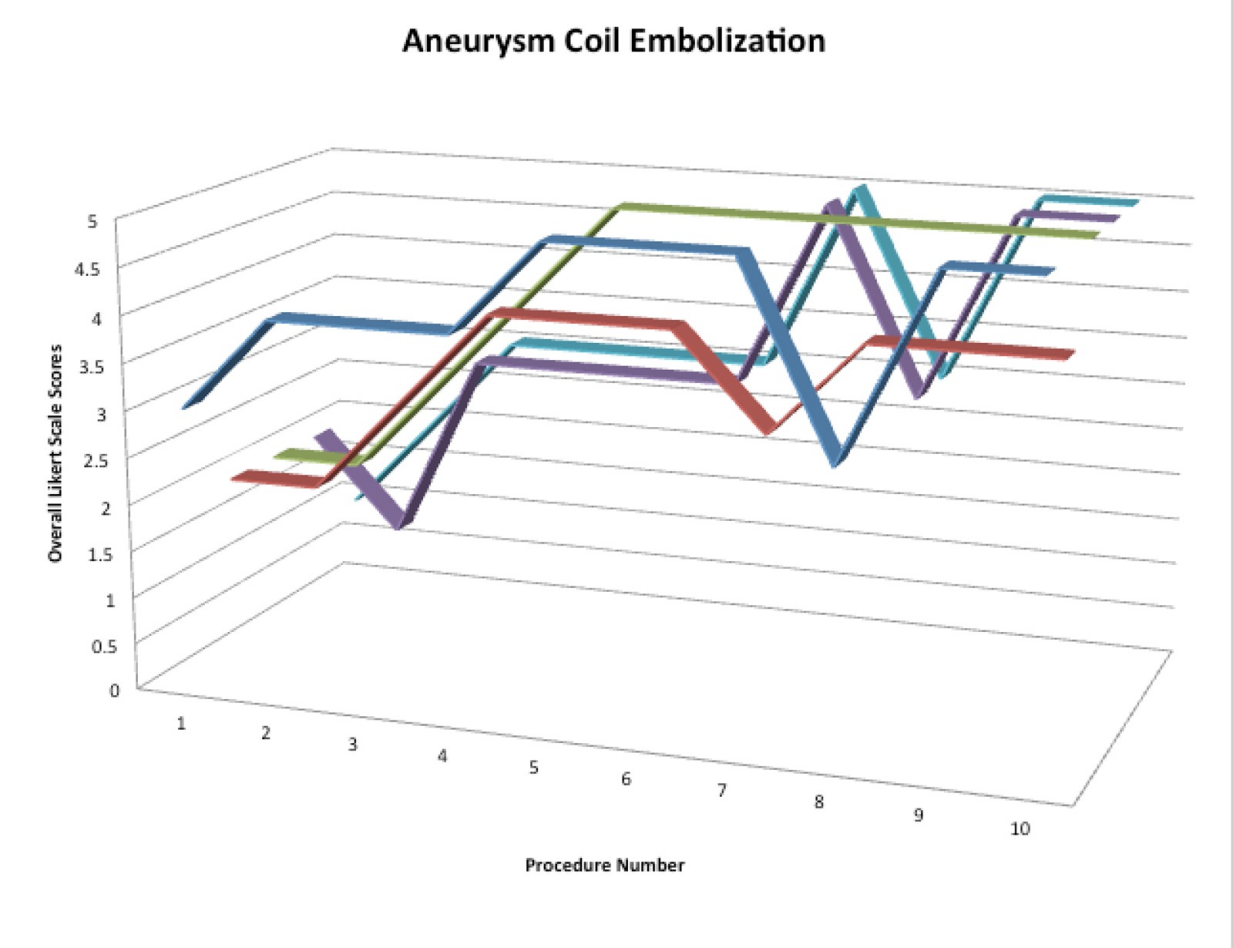
Color coded 3d line graph of overall Likert Scale performance scores Scores for each participant over the course of performing 10 permanent coil embolizations for aneurysm. Each participant is displayed in a different color.

After completion of the permanent aneurysm coil embolization portion of our simulator curriculum, participants demonstrated a 42% reduction in total procedure time, a 57% reduction in fluoroscopy time, and a 21% reduction in contrast utilization. There was no statistically significant difference in the quality of images produced during the coil embolization procedures. There was a reduction in unsafe techniques from an average absolute value of 6.4 events to 0 events. There was a 58% improvement in the overall Likert scale score (Figure 4).

All variables were again compared using the ANOVA and Tukey’s HSD tests to evaluate the significance of each variable between each procedure. Independent of prior experience, all participants demonstrated statistically significant reductions in procedure time, fluoroscopy time, contrast utilization, and the use of unsafe techniques during the first five procedures (p < 0.05 for all variables). During the last five procedures, all participants demonstrated a reduction in procedure time, fluoroscopy time, contrast utilization, and the use of unsafe techniques, but the differences were not statistically significant on the 95% confidence interval (average p = 0.118). The endovascular neurosurgical fellow demonstrated statistically significant improvements in Likert Scale scores up to and including the fifth procedure at which time the maximum score was obtained and maintained for the remainder of the exercise. However, the less experienced participants continued to experience intraprocedural aneurysm ruptures occurring due to the exertion of greater force than appropriate for coil placement up to and including the eighth procedure at which time the maximum score was obtained and maintained.

### Mechanical embolectomy

**Figure 5:**
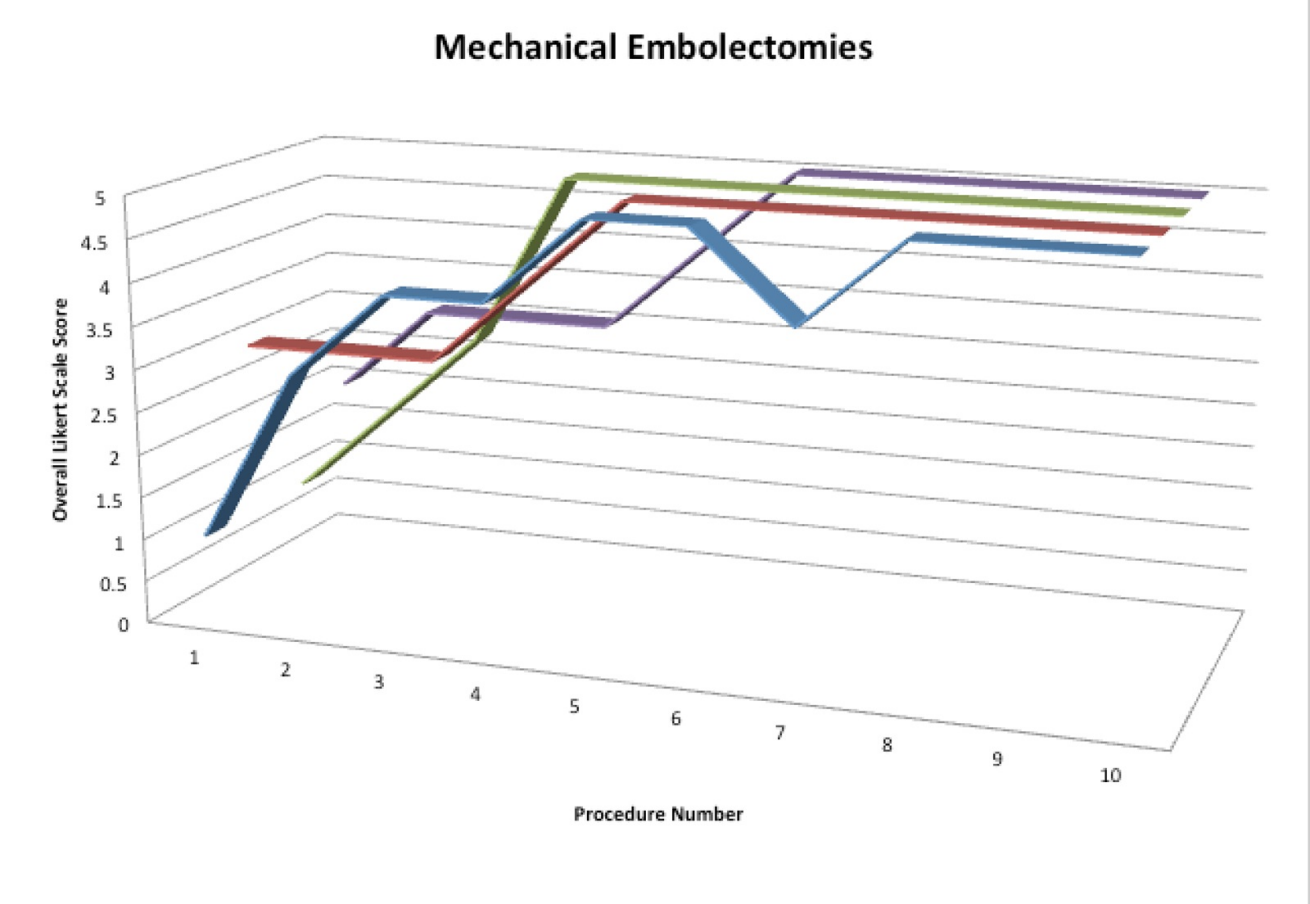
Color-coded 3d line graph of overall Likert Scale performance scores Scores for each participant over the course of performing 10 mechanical embolectomies. Each participant is displayed in a different color.

After completion of the mechanical embolectomy portion of our simulator curriculum, participants demonstrated a 35% reduction in total procedure time, a 41% reduction in fluoroscopy time, and a 49% reduction in contrast utilization. There was no statistically significant difference in the quality of images produced during the mechanical embolectomy procedures. There was a reduction in unsafe techniques from an average absolute value of 5.3 events to zero events. There was a 67% improvement in the overall Likert scale score (Figure 5).

All variables were compared using ANOVA and Tukey’s HSD tests to evaluate the significance of each variable between each angiogram.  Independent of prior experience, all participants demonstrated statistically significant differences in performance during the first five embolectomies (p < 0.05 for all variables). During the last five embolectomies, all participants demonstrated improvement, but the differences were not statistically significant on the 95% confidence interval (average p = 0.158).

## Discussion

Based upon the statistical analysis of the 30 separate parameters recorded during this simulation and the associated Likert scale scores, all participants demonstrated statistically significant performance improvements after performing five diagnostic cerebral angiograms, five mechanical embolectomies, and nine aneurysm coiling procedures. Therefore, based on our results, we would recommend that neurosurgical residents and neuroradiology fellows should perform a minimum of five simulated angiograms, five simulated embolectomies, and 10 simulated aneurysm permanent coil embolizations prior to scrubbing for endovascular neurosurgery cases. However, our recommendations are predicated upon adequate supervision during the simulated experience and achievement of minimum Likert scores of 4 or greater during the exercise.

The major limitations of this study are the small number of participants, the diversity of participants, the lack of a control group, and the exclusion of complex brachiocephalic anatomies, such as Type III and bovine arch types. Moreover, we did not include more complex procedures, such as stent-assisted coil embolization of more distal mechanical embolectomies like M2 segment occlusions. Finally, our performance metrics and scoring system have not yet been correlated with performance in the angiography suite. Future areas of investigation will incorporate larger groups of participants, more complex procedures, more complex access, and will include pre- and post-simulation assessments of clinical performance in the angiography suite.

## Conclusions

In summary, endovascular neurosurgical simulations are a useful education exercise offering a consequence and risk-free environment for training inexperienced neurosurgery fellows, neurointerventional radiology fellows, and interventional neurology fellows. In this study, we established an introductory simulator curriculum and statistically validated performance metrics. However, we recognize the need for future studies correlating our simulator curriculum with improvement in clinical angiographic and endovascular neurosurgical performance.
